# Sequence and phylogenetic analysis of the mitochondrial genome for red-breasted parakeet *Psittacula alexandri* (Psittaciformes: Psittacidae)

**DOI:** 10.1080/23802359.2019.1678417

**Published:** 2019-10-16

**Authors:** Yuan Li, Yubao Duan

**Affiliations:** a Key Laboratory for Forest Resources Conservation and Utilization in the Southwest Mountains of China, Ministry of Education, Southwest Forestry University, Kunming, Yunnan Province, China;; b Wildlife Conservation Institute, Central South University of Forestry and Technology, Changsha, Hunan Province, China;; c Key Laboratory for Conserving Wildlife with Small Populations in Yunnan, Southwest Forestry University, Kunming, Yunnan Province, China;; d Faculty of Biodiversity Conservation, Southwest Forestry University, Kunming, Yunnan Province, China

**Keywords:** *Psittacula alexandri*, mitogenome, phylogeny

## Abstract

In this study, we first sequenced and described the complete mitochondrial genome and phylogeny of *Psittacula alexandri*. The whole genome of *P. alexandri* was 16,883 bp in length, and contained 14 protein-coding genes, 22 transfer RNA genes, 2 ribosome RNA genes, and 1 non-coding control regions. The overall base composition of the mitochondrial DNA was 30.87% for A, 22.36% for T, 32.82% for C, and 13.95% for G, with a GC content of 46.77%. A phylogenetic tree strongly supported that *P. alexandri* closely related with *Eclectus roratus* by highly probability.

Red-breasted Parakeet (*P. alexandri* Linnaeus 1758) which belongs to the family Psittacidae of order Psittaciformes, mainly perched on a variety of forest and wooded habitats, including human-altered areas, usually below 2000 m (BirdLife International 2019). Its diet includes wild and cultivated fruits, berries, flowers, nectar, nuts and seeds, leaves and cereals such as rice and maize, thus it frequently causes damage to crops. In Nepal, the species is regarded to be easiest parakeet to catch for the pet-trade because of its flocking behaviour and relatively sluggish nature. *Psittacula alexandri* occurs in south and south-east Asia, the population number of the species showed a downward trend (BirdLife International 2019). It was listed as Near Threatened (NT) on the IUCN Red List (IUCN 2019). Astuti et al. (2006) revealed phylogenetic relationships within Parrots (Psittacidae) using mitochondrial cytochrome-b gene sequences. Very few studies on *P. alexandri* as a commonly known species had been reported about complete mitochondrial genome. Therefore, we sequenced the complete mitochondrial genome of *P. alexandri* to enhance our understanding on the phylogeny of Psittacidae.

The specimen was collected from Dehong Wildlife Rescue Centre (24°21′76″N, 98°28′84″E), which was located northwestern of Yunnan Province in China, and stored in Herbarium of Southwest Forestry University. A specimen Accession number is Duan-02. The total mitochondrial DNA was extracted from the muscle tissue using Next Generation Sequencing. The complete mitochondrial genome of *P. alexandri* was submitted to the NCBI database under the accession number MK986660. Phylogenetic tree of the relationships among Strigopidae, Cacatuidae, Psittacidae and outgroup were presented using 20 species using maximum likelihood (ML) methods available on the CIPRES Science Gateway v3.3 (Miller 2010). Bayesian inference was calculated with MrBayes3.1.2 with a general time reversible (GTR) model of DNA substitution and a gamma distribution rate variation across sites. Sequences of Columbiformes (*Columba livia* and *Spilopelia chinensis*) obtained from GenBank (NC_020424 and GU908131) were used as outgroups to root trees following Astuti et al. (2006).

The complete mitochondrial genome of *P. alexandri* was 16,883 bp in length. A total of 39 mitochondrial genes were identified, including 14 protein-coding genes (PCGs), 22 transfer RNA (tRNA) genes, 2 ribosomal RNA (rRNA) genes, and 1 non-coding control region (D-loop). Among these genes, *nad*6 and 8 tRNAs (*trnQ*, *trnA*, *trnN*, *trnC*, *trnY*, *trnS2*, *trnP*, and *trnE*) were located on the light strand (L-strand), while all of the remaining genes were located on the heavy strand (H-strand). The overall base composition of *P. alexandri* mitogenome was 30.87% for A, 22.36% for T, 32.82% for C, and 13.95% for G, A + T content is 53.23%, which is higher than G + C content of 46.77%, similar to other Psittaciformes (Sarker et al. 2018; Liu et al. 2019).

The reconstructed phylogenetic tree supported the placement of *P. alexandri* in the family Psittacidae of order Psittaciformes (Figure 1). Our results 18 species were clustered into three groups. *P. alexandri* closely related with *Eclectus roratus*, and was strongly supported by the analyses of protein-coding genes. Thus, the mitochondrial genome reported here would be useful in the current understanding of the phylogeny and evolution of Psittacidae.

**Figure 1.**
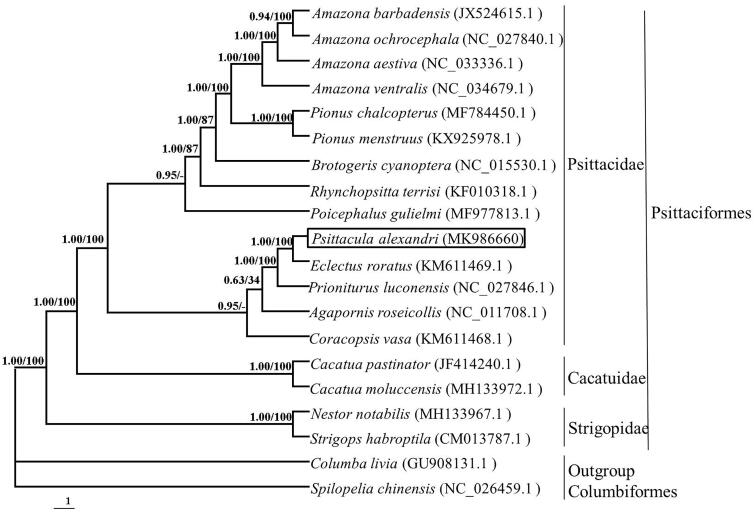
The phylogenetic tree based on combing protein-coding gene sequences of 20 species. Numbers at node of the tree branches represent Bayesian posterior probability (left) and RAxML rapid bootstrap support (right).

## References

[CIT0001] Astuti D, Azuma N, Suzuki H, Higashi S 2006 Phylogenetic relationships within parrots (Psittacidae) inferred from mitochondrial cytochrome-b gene sequences. Zool Sci. 23(2):191–198. 10.2108/zsj.23.191 16603811

[CIT0002] BirdLife International. 2019 Species factsheet: *Indicator xanthonotus* [accessed 2019 July 9]. http://www.birdlife.org.

[CIT0004] IUCN 2019 The IUCN Red List of Threatened Species. Version 2019-1. [accessed 2019 July 9]. www.incnredlist.org.

[CIT0005] Liu H, Jin K, Li L 2019 The complete mitochondrial genome of the Fischer's Lovebird *Agapornis fischeri* (Psittaciformes: Psittacidae). Mitochondr DNA B. 4(1):1217–1218.

[CIT0006] Sarker S, Das S, Ghorashi SA, Forwood JK, Helbig K, Raidal SR 2018 The first complete mitogenome of red-bellied parrot (*Poicephalus rufiventris*) resolves phylogenetic status within Psittacidae. Mitochondr DNA B. 3(1):195–197. 10.1080/23802359.2018.1437818 PMC7800220 33474115

